# Pseudo-Signet Ring Cells: Diagnostic Pitfalls—Insights from Case Reports

**DOI:** 10.3390/reports9020144

**Published:** 2026-05-05

**Authors:** Lina Chen, Lai Mun Wang, Runjan Chetty, Sangeetha N. Kalimuthu

**Affiliations:** 1 Anatomic Pathology, Precision Diagnostics and Therapeutics Program, Sunnybrook Health Sciences Centre, 2075 Bayview Ave, Room E420A, Toronto, ON M4N 3M5, Canada; 2 Department of Laboratory Medicine and Pathobiology, University of Toronto, Toronto, ON M5S 1A1, Canada; 3 Department of Laboratory Medicine, Changi General Hospital, Singapore 529889, Singapore; 4 Department of Pathology, Laboratory Medicine Program, University Health Network, University of Toronto, Toronto, ON M5S 1A1, Canada

**Keywords:** pseudo-signet ring cell, signet ring cell adenocarcinoma

## Abstract

**Background:** The term “pseudo-signet ring cell” in the gastrointestinal and biliary tract refers to benign cells with signet ring-like morphology that resemble the malignant counterpart seen in poorly differentiated adenocarcinomas; **Clinical Significance:** Given this close resemblance to malignant cells, they can pose a diagnostic challenge for pathologists. Awareness of this diagnostic pitfall is crucial to avoid misdiagnoses and overtreatment of patients; **Case Presentation:** Herein, we provide an overview of an array of clinical presentations of pseudo-signet ring cells, particularly focusing on the three most frequent clinical scenarios, and briefly discuss the possible etiologies for this phenomenon; **Conclusions:** Pseudo-signet ring cells are a rare but important diagnostic pitfall that require careful morphological evaluation, contextual awareness, and clinicopathologic correlation to avoid misdiagnosis.

## 1. Introduction

Signet ring cells are round cells with cytoplasmic mucin and a peripherally displaced crescent-shaped nucleus [1], morphologically resembling a signet ring. This appellation is usually applied to poorly differentiated adenocarcinomas that have signet-ring morphology. Signet ring cell adenocarcinoma (SRCA) is an aggressive malignancy with a poor prognosis and occurs most frequently in the gastrointestinal and biliary tracts.

However, in certain clinical circumstances, cells with similar morphology, such as benign mucin-containing epithelial cells, non-mucin containing epithelial cells, and cells of non-epithelial origin, mimic the malignant signet ring carcinoma cells. Such cytological changes are referred to by a spectrum of monikers in the literature, including signet-ring cell change or signet ring-like cells [1,2,3,4]. However, the aforementioned terminology carries the risk of misinterpretation clinically. Accordingly, the term pseudo-signet ring cells (PSRCs) is now preferred to emphasize the non-neoplastic or reactive nature of this phenomenon. The underlying etiology and pathogenesis of PSRCs remain unclear, although reactive or degenerative changes as a result of ischemia, inflammation, or ulceration have been hypothesized [1,5].

Failure to recognize PSRCs represents an important diagnostic pitfall and may result in misdiagnosis as SRCA, potentially leading to unnecessary aggressive treatment such as surgery or chemoradiation. Avoiding this pitfall requires awareness of the clinical settings in which PSRCs occur and careful assessment of histomorphologic features that distinguish them from true carcinoma. While ancillary studies can be helpful in selected cases, morphologic evaluation in conjunction with clinical context remains the cornerstone of diagnosis.

This study represents a descriptive, scenario-based series combined with a narrative review of the literature. The scenarios were identified from routine diagnostic material encountered in gastrointestinal pathology practice at tertiary academic centers and were selected because they reflect common diagnostic challenges involving signet ring–like morphology in benign or non-neoplastic conditions. A targeted literature review was performed to summarize reported morphologic features, diagnostic pitfalls, proposed etiologies, and the role of ancillary studies.

This work is intended to highlight practical diagnostic challenges and to provide a framework for recognizing pseudo-signet ring cells in routine practice.

## 2. Scenario Presentations

### 2.1. Scenario 1. Ischemic Small Bowel Epithelial Cells

**Figure 1:**
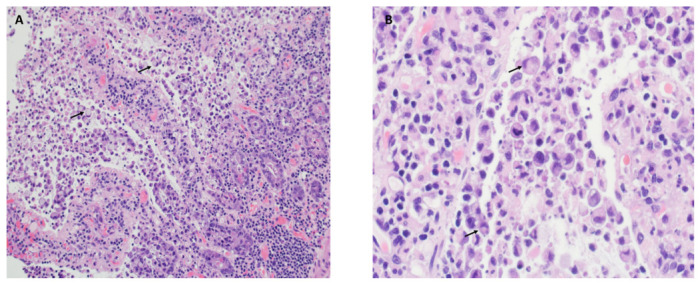
Pseudo-signet ring cells in ischemic small bowel (**A**,**B**). (**A**). Small bowel biopsy shows superficial epithelial necrosis with atypical epithelial cells (H&E, 20×). (**B**). The atypical epithelial cells in the superficial part of the mucosa have signet ring-like morphology (H&E, 40×). Arrows point to a few cells with classical signet ring-like morphology.

An elderly male presented with acute abdominal pain and underwent small bowel resection for ischemia. The sections showed superficial epithelial necrosis with preservation of deep crypts, villous distortion, ulceration, and surface exudate of neutrophils and fibrin (Figure 1A). Incidentally, atypical epithelial cells with signet ring-like morphology were found extensively in the superficial part of the mucosa (Figure 1A,B). They were discohesive and had abundant intracytoplasmic mucin. However, they were confined to the superficial mucosa, and the associated mucosa was ulcerated or degenerative. Although there was mild cytological atypia, given the clinical history and the absence of these cells within the more viable and intact mucosa, these were PSRCs.

### 2.2. Scenario 2. Degenerative Gastric Epithelial Cells Due to PPI Effect

**Figure 2:**
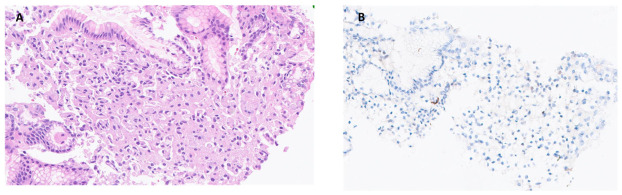
Pseudo-signet ring cells in gastric mucosa associated with proton pump inhibitor use. (**A**). Stomach biopsy shows the epithelial cells of the fundic glands having signet ring-like morphology without any cytological atypia (H&E, 20×). (**B**). P53 is wild type (P53 immunostain, 20×).

A middle-aged female presented with dysphagia and had been using a proton pump inhibitor (PPI) for a few months. She underwent an upper gastroendoscopy, which showed no endoscopic abnormality, and routine biopsies of the gastric antrum and body were taken. The epithelial cells of the fundic glands showed signet ring-like morphology (Figure 2A). They were confined within the glands without any invasive growth and did not demonstrate any cytological atypia. P53 is wild type (Figure 2B), and the Ki67 proliferative index is very low (approximately 1–2%). Overall, the bland cytological features combined with the IHC results excluded the diagnosis of a true SRCA.

### 2.3. Scenario 3. Non-Epithelial Atrophic Adipocytes

**Figure 3:**
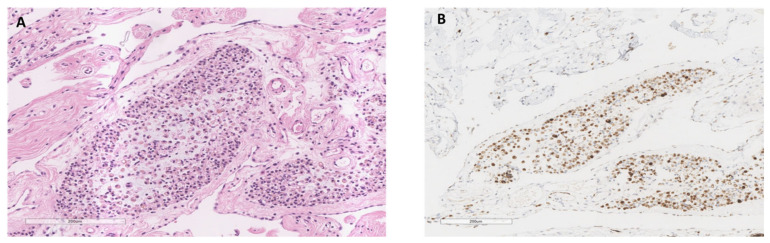
Atrophic adipocytes mimicking signet ring cells in mesenteric fat. (**A**). The subtotal colectomy specimen shows the presence of multiple lobules of signet ring-like cells within the mesenteric fat (H&E, 10×). (**B**). IHC shows S100 positive (S100 immunostain, 5×).

An elderly male with significant unintentional weight loss (more than 20 kg over a year) presented with sigmoid perforation and underwent subtotal colectomy. On histological examination, the striking feature was the presence of multiple lobules of signet ring-like cells within the mesenteric fat (Figure 3A). These cells were smaller than the typical mature adipocytes and demonstrated minimal pleomorphism. The clear cytoplasmic vacuole pushed the round to oval-shaped nucleus to the side. There was no evidence of nuclear atypia or mitosis. The remaining cells maintained the typical configuration of adipocytes. Some were a little hyperchromatic; however, they had no nuclear indentation or scalloping. They had thickened cell membranes, surrounded by myxoid stroma with a delicate fibrovascular network. IHC showed S100 positive (Figure 3B), confirming adipocytic lineage.

## 3. Discussion

In the above cases, we demonstrated the striking morphologic resemblance between PSRCs and signet ring cell carcinoma and highlighted the diagnostic challenges they pose. The failure to recognize this phenomenon can lead to a diagnostic pitfall.

Ischemia-associated PSRCs have been reported throughout the gastrointestinal tract and are typically confined to the superficial mucosa in areas of ulceration [1,5,6,7,8]. They do not show any significant cytological atypia. However, subacute ischemic changes have been reported to closely mimic infiltrating diffuse-type signet ring cell carcinoma [8]. These PSRCs are located in the deeper aspects of the necrotic/degenerative mucosa but not found in the viable mucosa.

PSRCs have also been reported in other reactive/inflammatory/infectious conditions in the GI tract: pseudomembranous colitis (C. diff), ulcerative colitis, cholangitis, cholecystitis, and ulceration [2,3,4,5,6,7,9,10,11,12,13,14,15,16,17,18]. In pseudomembranous colitis, the incidence of PSRCs can be up to 28% and most of those do not have an infiltrative pattern [16,19]. However, in ulcerative colitis, extensive infiltrative-appearing PSRCs have been reported [9]. In these scenarios, P53 and Ki67 can be used to exclude malignancy. PSRCs in the biliary tract are found with acute or erosive cholangitis or cholecystitis [2,5,12,15]. They can be found in deep mucosal glands and can exhibit a pseudo-infiltrative pattern due to adjacent ulceration or necrotic tissue, but they are always confined to the basement membrane of the glands. Stains for the basement membrane, such as PAS, anti-collagen IV, and reticulin, may be used to identify the basement membrane layer.

PSRCs caused by long-term PPI use, as demonstrated earlier, have been reported and can mimic signet ring cell carcinoma in situ [6]. Long-term PPI use promotes parietal cell hyperplasia and vacuolization. Some of these cells, with extensive vacuolization, can have eccentric crescent-shaped nuclei. These cells are positive for mucin stain and considered degenerative fundic gland cells. Therefore, distinguishing them from signet ring cell adenocarcinoma can be difficult. Morphologic features that have been associated with signet ring cell adenocarcinoma include an infiltrative growth pattern, cellular atypia, nuclear hyperchromasia, prominent nucleoli, and mitoses. IHC for intact basement membrane and Ki67 can be used for diagnosis [6,7]. However, signet ring cell adenocarcinoma in situ can have no invasion and may harbor deceptively bland cytological features. Moreover, PSRCs could have mitotic activity due to regeneration [7]. A normal E-cadherin expression pattern and a lack of p53 staining in the cells with signet ring morphology support the diagnosis of PSRCs [7].

PSRCs can also be seen with neuroendocrine cell hyperplasia [20,21]. PPI causes neuroendocrine hyperplasia, and some of these neuroendocrine cells have clear cell change mimicking signet ring cells. The long-term use of PPIs has been suggested to be associated with a higher risk of gastric cancer development [22]. ECL cells may lose many of their neuroendocrine characteristics during neoplastic transformation [23,24]. Some studies suggest that occasional gastric adenocarcinomas, in particular the signet ring subgroup of diffuse-type gastric adenocarcinomas, potentially develop from ECL cells [23,24,25]. This makes the diagnosis very challenging. In these cases, mucin stains and neuroendocrine markers are not useful in differentiating between PSRCs and malignant signet ring cells. As such, recognizing the key morphological features of PSRC, particularly their confinement to the superficial glands without invasion of lamina propria, is essential to distinguish the benign from the malignant entity [20,21].

Dystrophic adipocytes mimicking signet ring cells have been reported in both animal models and humans [26,27,28]. They are found in cachexia with profound loss of adipose tissue due to different reasons such as starvation, malignancy, and systemic disease. As demonstrated in our case above, they are characterized by small signet ring-like cells arranged in a non-infiltrative lobular pattern in a mucoid/myxoid stroma with a delicate capillary network. They can sometimes also mimic lipoblasts and thus be misdiagnosed as myxoid liposarcoma. However, the location, lobular configuration and lack of mass lesions favor a benign process. IHC may be utilized to confirm the lineage, if necessary.

Cells with signet ring-like morphology can be recognized in other benign/malignant neoplasms of the GI tract, such as tubular adenoma, Peutz–Jeghers polyp, neuroendocrine tumors, lymphoma, gastrointestinal stromal tumors, neurofibroma and schwannoma [29,30,31,32,33]. They can be derived from epithelial or non-epithelial cells. These PSRCs are not necessarily benign; for example, PSRCs in neuroendocrine tumors are malignant [34]. Although rarely reported as primary GI tract tumors, myxoid liposarcoma, fat-poor angiomyolipoma, and clear cell sarcoma-like tumors can also mimic signet ring cell carcinoma [35,36,37,38,39]. The treatment and prognosis of these conditions are very different from those of signet ring cell carcinoma. Awareness of the existence of PSRCs in these conditions may help avoid misdiagnosis of primary or metastatic signet ring cell carcinoma.

Another form of PSRCs that should be recognized is mucin-producing mesothelial cells [40]. Mesothelial markers are very useful when pathologists encounter these changes histologically. PSRCs are rare in non-epithelial cells that are not associated with neoplasms [41]. In addition to the atrophic adipocytes presented above, other non-neoplastic non-epithelial PSRCs include mucophagocytizing histiocytes and xanthomas [42,43]. Ancillary studies would be useful in determining the lineage origin of these cells.

**Figure 4:**
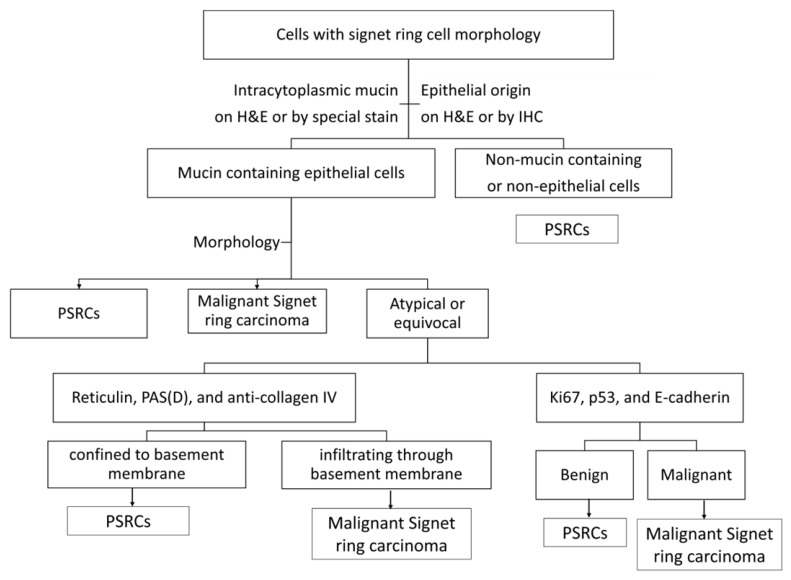
Diagnostic flowchart for cells with signet ring cell morphology. Proposed diagnostic flowchart outlining a practical, stepwise approach to evaluating cells with signet ring–like morphology, emphasizing the integration of morphology, architectural context, clinical history, and judicious use of ancillary studies to distinguish pseudo-signet ring cells from true signet ring cell carcinoma.

**Figure 5:**
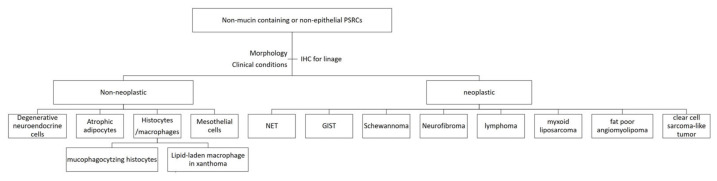
Summary for non-mucin-containing or non-epithelial PSRCs. Schematic summary of non-mucin-containing and non-epithelial entities that may demonstrate pseudo-signet ring cell morphology.

Although it is not essential to use special stains and IHC in cases of PSRCs, they do serve as a useful adjunct to the morphological features and can help alleviate the diagnostic conundrum. To begin, the general approach would be to determine if they are mucin-containing, using mucicarmine and PASD. Next, cytokeratin, CD68, S100, and neuroendocrine markers can be used for identifying the lineage in the appropriate context. In mucin-containing epithelial cells, Ki67, p53, and E-cadherin can be useful to exclude malignancy. Reticulin, PAS(D), and anti-collagen IV can help determine the confinement within the basement membrane. However, pathologists should keep in mind that these ancillary tests are of limited utility. Clinical correlation and morphological features still remain the benchmark for recognizing this entity. The flowcharts in Figure 4 and Figure 5 further summarize the above approach.

The exact etiology and mechanism of PSRC are still not clear. For epithelial PSRCs, some have proposed ischemia as the etiologic origin [5,8]. In ischemic colitis, the PSRCs express epithelial markers that are derived from goblet cells [44]. Goblet cells are mucin-producing cells that have a protective function in bowel ischemia and reperfusion injury [8,44]. The signet ring-like morphological change is thought to be an adaptive proliferative response [8]. For non-epithelial PSRCs, they are mostly associated with reactive/degenerative processes, but the exact mechanisms depend on the lineage they are derived from [27,28,41,42,45]. For atrophic fat PSRCs, it has been proposed that cachexia-induced impairment in lipid formation and storage capacity contributed to the morphological changes in adipose tissue [26]. In neoplastic conditions, the pathogenesis of cells with signet ring-like morphology depends on the tumor type and PSRC cell type, such as the accumulation of neurosecretory granules and myelin in neuroendocrine tumors and glycogen accumulation in GIST [32,34].

In conclusion, pseudo-signet ring cells represent an uncommon but clinically important diagnostic pitfall. Careful assessment of morphology, attention to architectural context, and clinicopathologic correlation remain paramount, particularly in settings with limited access to ancillary testing. Awareness of this phenomenon is essential to prevent overdiagnosis and inappropriate aggressive therapy.

## Data Availability

The original contributions presented in this study are included in the article. Further inquiries can be directed to the corresponding author.

## Abbreviations

The following abbreviations are used in this manuscript:

SRCA	signet ring cell adenocarcinoma
PSRC	pseudo-signet ring cell
PPI	proton pump inhibitor
